# lncRNA VIM-AS1 acts as a prognostic biomarker and promotes apoptosis in lung adenocarcinoma

**DOI:** 10.7150/jca.83639

**Published:** 2023-05-15

**Authors:** Jianhong Kang, Maimaiti Abudurufu, Shuwei Zhang, Wei Jiang, Honghe Luo

**Affiliations:** 1 Department of Thoracic Surgery, The first Affiliated Hospital of Sun Yat-sen University, Guangzhou, Guangdong, China.; 2 Department of Thoracic Surgery, Sichuan Academy of Medical Sciences and Sichuan Provincial People's Hospital, Chengdu, Sichuan, China.; 3 Anesthesia Surgery Center, Sichuan Academy of Medical Sciences & Sichuan Provincial People's Hospital, Chengdu, Sichuan, China.

**Keywords:** long non-coding, VIM-antisense 1, lung adenocarcinoma, prognosis, apoptosis

## Abstract

**Background:** Long non-coding RNA VIM-antisense 1 (VIM-AS1) has been reported that it is involved in the progression of several cancers. However, the aberrant expression profile, clinical significance, and biological function of VIM-AS1in lung adenocarcinoma (LUAD) have not been fully described. We tend to perform a comprehensive analysis to identify the clinical prognostic value of VIM-AS1 for LUAD patients and explore its potential molecular mechanisms in LUAD development.

**Methods**: The expression features of VIM-AS1 in LUAD were identified based on Cancer Genome Atlas database (TCGA) and genotypic tissue expression (GTEx). The LUAD patients' lung tissues were collected to testify above expression features. Survival analysis and COX regression analysis were performed to evaluate the prognostic value of VIM-AS1 in LUAD patients. Then Correlation analysis was performed to filter VIM-AS1 co-expression genes, and their molecular functions were constructed. Furtherly, we constructed the lung carcinoma A549 cell line with VIM-AS1 overexpression to test its effect on cell function.

**Results**: VIM-AS1 expression levels were significantly downregulated in LUAD tissues. VIM-AS1 with low expression is significantly associated with short overall survival (OS), disease-specific survival (DSS), progress free interval (PFI), late T pathological stage, and lymph node metastasis for LUAD patients. The low expression level of VIM-AS1 was an independent risk factor for poor prognosis for LUAD patients. The biological functions of co-expressed genes indicated that VIM-AS1 regulating the apoptosis process may be the potential mechanism for LUAD. Specifically, we testified VIM-AS1 can promote apoptosis in A549 cells.

**Conclusion**: VIM-AS1 was significantly downregulated in LUAD tissues, and it can be a promising prognostic index for LUAD development. VIM-AS1 regulating apoptotic effects may play important roles in LUAD progression.

## Introduction

Lung cancer is regarded as a common malignant tumor, and it is the leading cause of cancer-related death worldwide. Despite various treatments applied to diagnose and treat lung cancer, the five-year survival rate of lung cancer remains poor 1. With the rapid evolution of genome‑wide and transcriptome sequencing technologies, the functions of long non‑coding RNAs (lncRNAs) have received increasing attention 2. lncRNAs are a kind of RNA whose transcripts with a length of more than 200 nucleotides, which have no protein coding function due to a lack of a complete open reading frame 3. Previous studies have confirmed that lncRNAs exert their effects by binding to DNA, mRNA, microRNA, and proteins, which play a crucial role in regulating chromatin status, gene activity, and gene expression at the transcriptional, post-transcriptional, translational, and post-translational levels 4. Several studies have shown that the lncRNA is diverse and complex in lung adenocarcinoma (LUAD) development 5. Moreover, it has been reported that several lncRNAs are the key factors that control the development and progression of lung cancer. Therefore, exploring the mechanisms and processes of lung adenocarcinoma from the perspective of studying lncRNA can provide richer information for the treatment of LUAD patients. Although a variety of lncRNAs have been found significant roles in the mechanisms of lung cancer, it should not be ignored that lncRNAs are variable and complex. Thus, it is necessary to explore more diverse lncRNA mechanisms in lung cancer.

In recent studies, the important biological functions of lncRNA VIM-antisense 1 (VIM-AS1) have been noticed in the progression of some cancers. For example, VIM-AS1 with high expression in bladder cancer was detected, which is associated with bladder cancer metastasis 6. VIM-AS1 could facilitate the epithelial mesenchymal transition (EMT) process and enhances metastasis in prostate cancer 7. VIM-AS1 also was found that highly expressed in high-grade lymph node metastasis and vascular invasion colorectal cancer 8. In contrast, VIM-AS1 plays the opposite effect on brain glioma. Inhibition of VIM-AS1 expression can inhibit glioma growth, colony formation, and migration 9.

**Figure 1:**
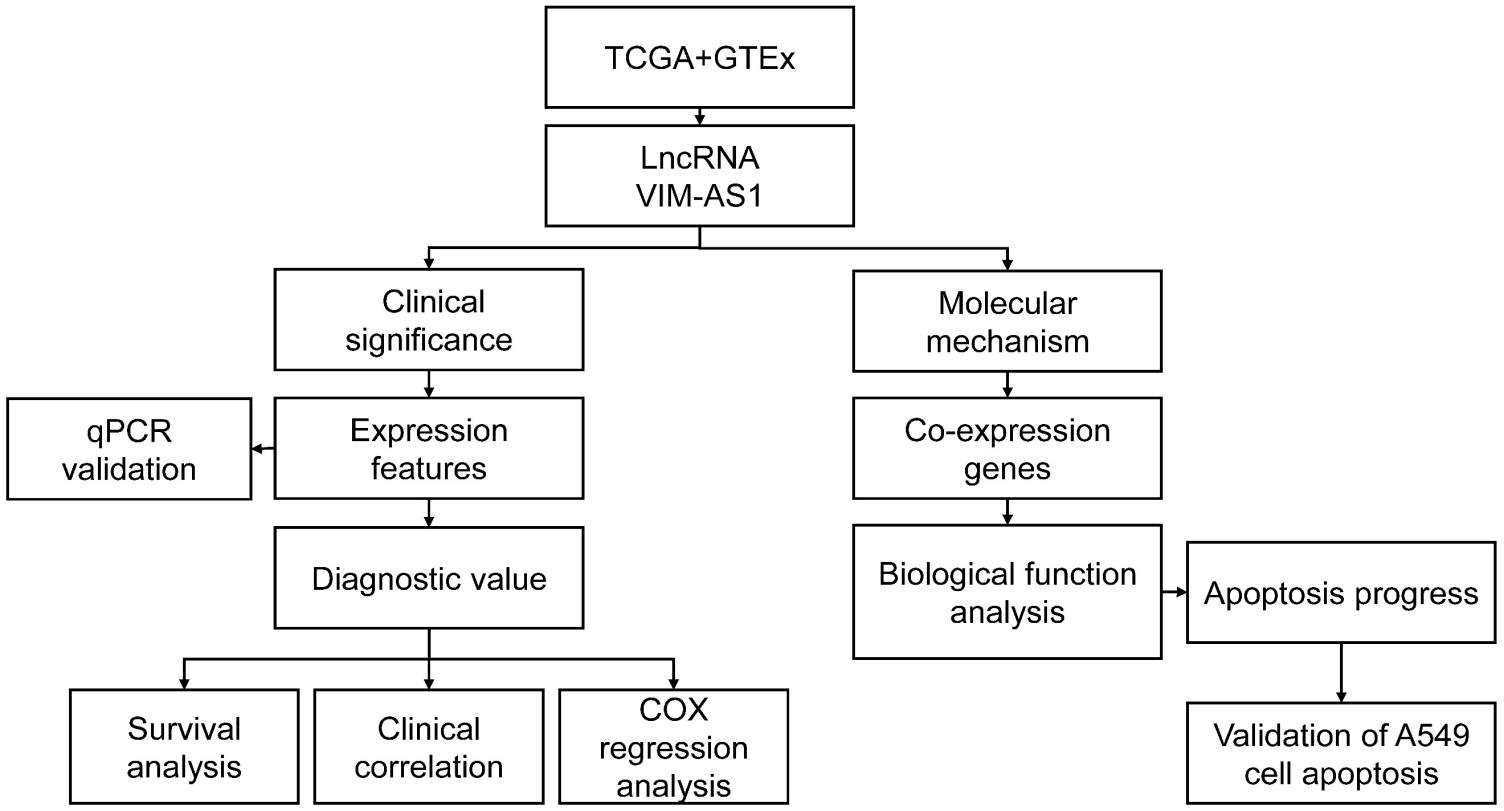
Flow chart of data analysis. TCGA: the cancer genome atlas, GTEx: the genotype-tissue expression, LncRNA: long non-coding RNA, VIM-AS1: vimentin antisense RNA1, qPCR: quantitative rea-time PCR, COX: cox proportional-hazards model.

Even if VIM-AS1 is considered to be a promising prognosis factor in different tumors, its clinical prognostic value in lung adenocarcinoma is barely known, and its relevant biological function is also fully understood. Therefore, this study proposed to explore the expression features of VIM-AS1 in LUAD tissues by bioinformatics analyses and experimental verification. We tend to identify the relationship between VIM-AS1 expression features and the clinicopathological characteristics, as well as prognostic value in LUAD. Furtherly, based on bioinformatics clues of VIM-AS1 in LUAD, we hope to explore the key molecular mechanisms of VIM-AS1 in LUAD progression (**Figure 1**).

## Materials and Methods

### Identification of the expression level of VIM-AS1 in LUAD tissues

Gene expression levels of LUAD patients were obtained from Cancer Genome Atlas database (TCGA) and genotypic tissue expression (GTEx). The transcriptomic data were TPM types. VIM-AS1 expression data were extracted by Perl language, then the difference of VIM-AS1 expression levels between normal and LUAD tissues was identified. Patients' normal lung tissue and LUAD tissue samples were paired to identify whether there was a difference in the expression levels of VIM-AS1.

### Identification of prognostic and clinicopathological characteristics of LUAD patients

LUAD patients were grouped according to the median VIM-AS1 expression values from TPM data. Kaplan-Meier (K-M) survival analysis was performed to identify the relationship between VIM-AS1 expression levels with prognostic characteristics, such as overall survival (OS), disease-specific survival (DSS), and progress free interval (PFI) in LUAD patients. We also investigated the relationship between expression levels of VIM-AS1 and the clinical characteristics (T-stage, N-stage, distant metastasis, pathological stage, age, gender) of LUAD patients.

### Identification of the relationship between VIM-AS1 expression levels and prognosis among subgroups of LUAD patients

Based on the data of clinical characteristics, such as T-stage, N-stage, distant metastasis, pathological stage, age, gender, and smoking history of LUAD patients. The median value of VIM-AS1 expression with TPM data was used to allocate LUAD patients. The relationship between VIM-AS1 expression level and the prognostic characteristics of subgroup LUAD patients was analyzed by K-M survival analysis.

### RNA extraction and quantitative real-time PCR (qPCR)

The lung tissues were obtained from LUAD patients at the first affiliated hospital of Sun Yat‐sen University between September 2003 and June 2015. This research was approved by the Ethics Committee and Institutional Review Board of Sun Yat‐sen University. Total RNA was extracted from lung adenocarcinoma tissues and paracancerous tissues, and A549 cells using the RNA simple Total RNA Kit (Tiangen) according to the manufacturer's instructions. A total of 1 µg RNA was used for RT, which was performed using the HiScript II Q RT SuperMix for qPCR (Vazyme), following the manufacturer's instructions. qPCR was performed using SYBR Green Master Mixture (Vazyme). The qPCR program was as follows: 95˚C for 5 min, followed by 40 cycles at 95˚C for 10 sec, 60˚C for 30 sec, 95˚C for 15 sec, 60˚C for 60 sec, 95˚C for 15 sec. GAPDH was used as a standardized internal control. The primer sequences were as follows: VIM-AS1 forward, 5'‑GACAGCAAAGCTCCCTTTGGA‑3' and reverse, 5'‑CCCGACGTGTTGTCCTGATG‑3'; and GAPDH forward, 5'‑AACGGATTTGGTCGTATTGGG‑3' and reverse, 5'‑CCTGGAAGATGGTGATGGGAT‑3'.

### Cox analysis and construction of VIM-AS1 nomogram

Univariate and multifactorial COX regression analyses were performed to analyze the relationship between T-stage, N-stage, distant metastasis, pathological stage, age, gender, smoking history, and VIM-AS1 expression levels with OS, DSS, and PFI in LUAD patients. *p*-value < 0.05 was the criterion for significance. Based on the multivariate Cox regression analysis results, nomogram was established to predict the prognosis of LUAD patients. The accuracy estimation of nomogram prediction was visualized in a calibration plot. The nomogram discrimination was determined using a concordance index, and 1,000 resamples were calculated by the bootstrap approach.

### Biological functions of VIM-AS1 co-expressed genes

Correlation analysis was performed to obtain VIM-AS1 co-expressed genes in LUAD tissues, and the absolute value of the correlation coefficient of 0.4 and *p*-value < 0.001 were used as the filter condition, and this condition was defined as VIM-AS1 significantly associated co-expressed genes. The Gene Ontology (GO) 10 was used to enrich the biological processes (BP), cellular components (CC), and molecular functions (MF) of these co-expressed genes.

### Culture and Construction of VIM-AS1 overexpress cell line

A549 cell lines were purchased from the American Type Culture Collection company (ATCC, Manassas, VA). A549 cells were cultured in DMEM media (Gibico) and supplemented with 10% fetal bovine serum (FBS) and 1% penicillin/streptomycin. Cells were maintained in an environment with 5% CO_2_ and 37 °C. The expression vectors containing VIM-AS1 were transfected into A549 cells. The sequence of VIM-AS1 was inserted in pPB-EF1a vector (Haixing Biosciences, China), while blank vector as control was transfected by using Lipofectamine® 2000 (Invitrogen; Thermo Fisher Scientific, Inc.), according to manufacturer's protocol.

### Test the effect of VIM-AS1 on apoptotic process

The A549 cells were digested with 0.25% EDTA‑free trypsin, then the Binding Buffer (Fcmacs Biotech suspended cells were added. Then, the cell suspension was transferred into a new tube. Then, Annexin V‑APc and PI (Fcmacs Biotech) were added, and the cells were incubated for 15 min at room temperature in the dark. Subsequently, apoptotic cells were detected using flow cytometry (BD Biosciences) and analyzed using FlowJo 7.6.1.

### Statistical analysis

The expression of VIM-AS1 in LUAD tissues was investigated by the Wilcoxon rank sum test, and prognostic factors in LUAD patients were assessed through K-M survival analysis and COX analysis. Comparisons between the two groups were performed using unpaired Student's t test. Correlation analysis was performed to identify the relationship between VIM-AS1 co-expressed genes in LUAD. *P* <0.05 was the criterion for the significance of this study.

## Results

### VIM-AS1 expression level was downregulated in LUAD tissues

**Figure 2:**
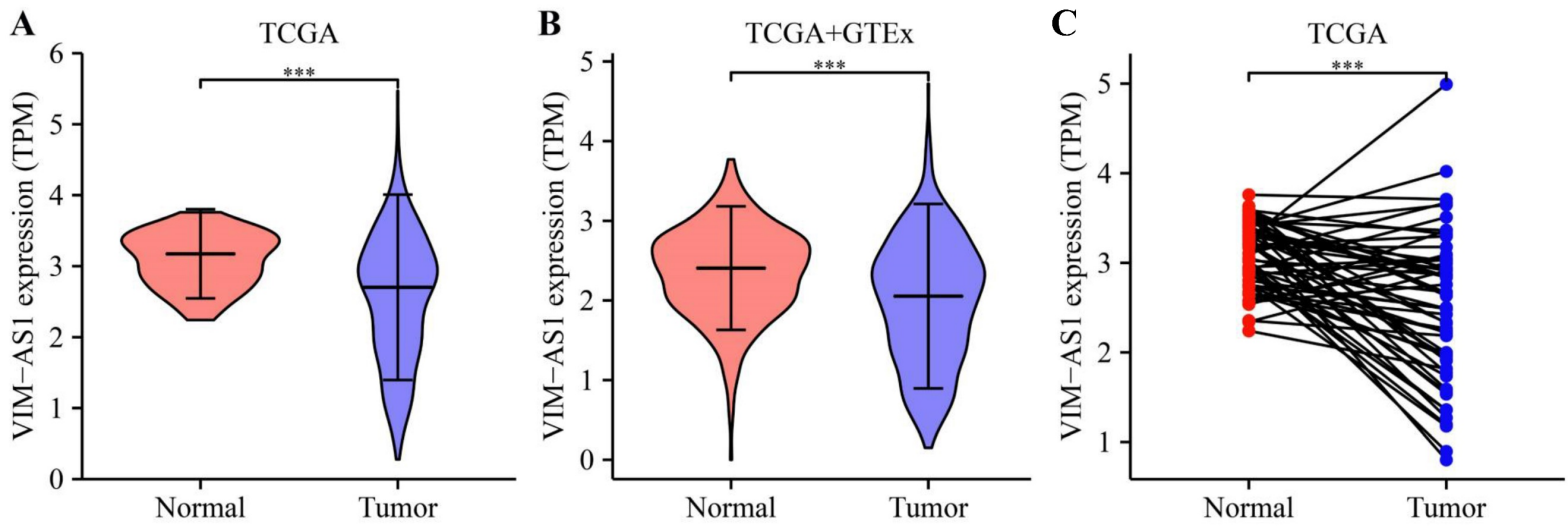
Expression feature of VIM-AS1 in lung cancer. **(A-B)** Expression of VIM-AS1 based on TCGA and GTEx dataset. **(C)** Expression levels of VIM-AS1 in paired adjacent normal tissues and paired samples. ***P<0.001.

Based on TCGA and GTEx databases, we simultaneously analyzed the expression profiles of VIM-AS1. Results showed that VIM-AS1 was expressed with low levels in lung adenocarcinoma compared to the normal samples (**Figure 2A-B**). The pairs of lung adenocarcinoma cancer samples and matched adjacent normal samples were obtained from TCGA data. We found that the expression of VIM-AS1 was decreased in lung adenocarcinoma samples than in matched adjacent normal samples (**Figure 2C**).

### Validation VIM-AS1 expression features in LUAD patients

**Figure 3:**
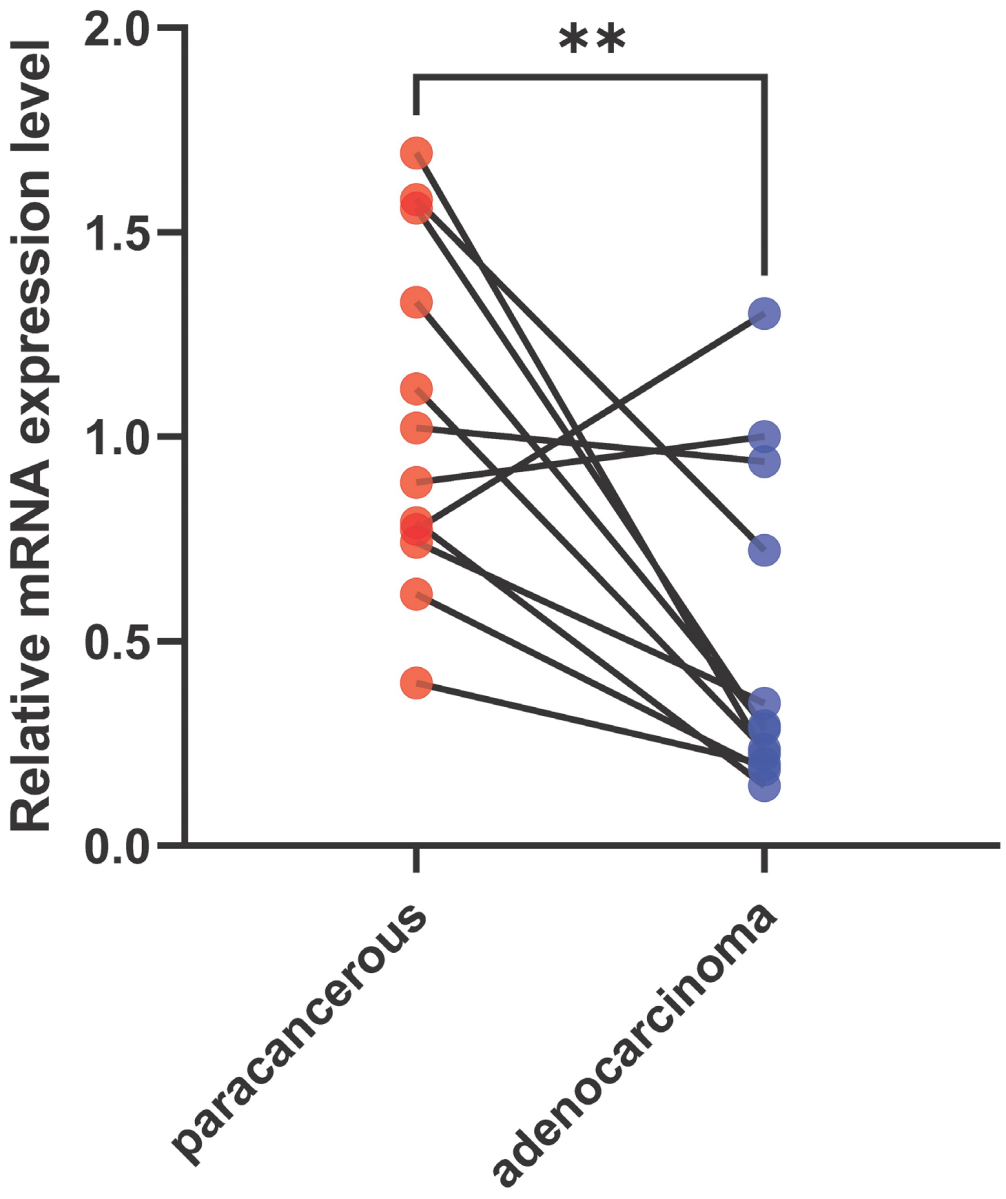
Expression levels of VIM-AS1 were assessed using qPCR. **P<0.01.

To validate the expression level of VIM-AS1, we collected lung adenocarcinoma tissues and paracancerous tissues from LUAD patient, and their clinical information are listed in **Table S1**. These tissues contain acinar adenocarcinoma lung and papillary lung adenocarcinoma, which are common lung adenocarcinoma types. We found the VIM-AS1 expression level was significantly lower than matched paracancerous tissues (*P*<0.05) (**Figure 3**). These results accord with above transcriptomic analyses.

### The low expression level of VIM-AS1 was correlated with poor clinical prognosis in LUAD patients

**Figure 4:**
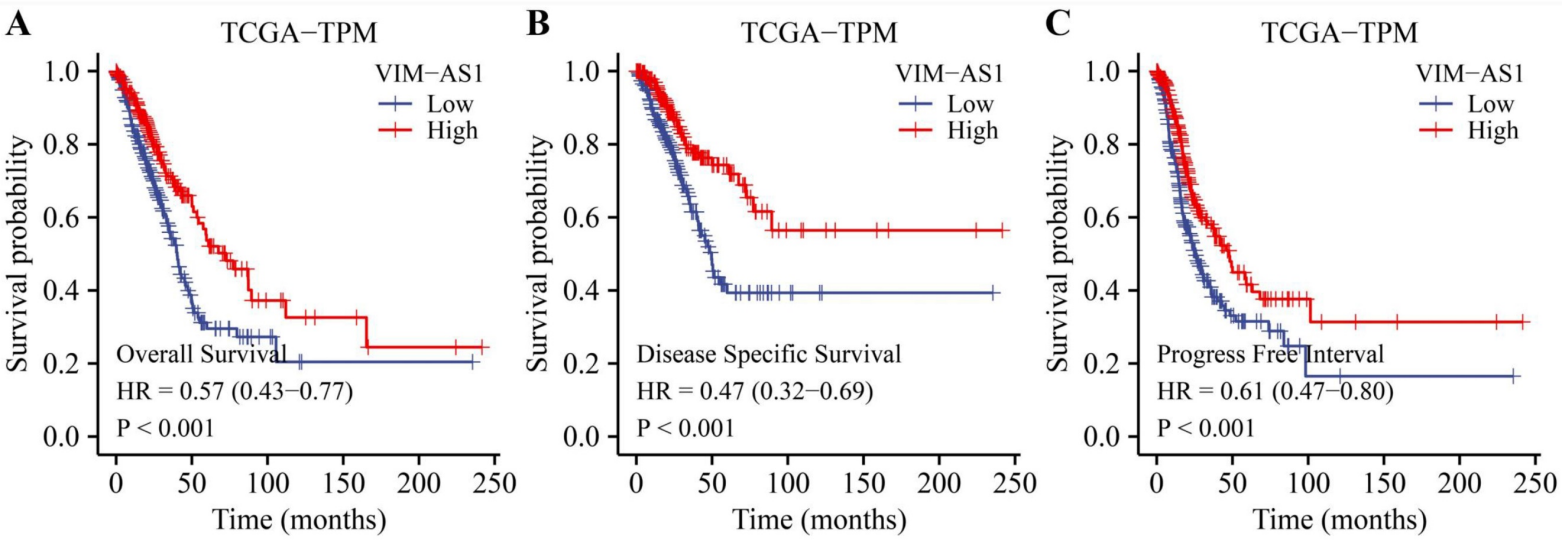
Kaplan-Meier survival curves showed that low VIM-AS1 expression levels in LUAD patients are associated with poor overall survival OS **(A)**, disease-specific survival DSS **(B)**, and progression-free survival (PFI) **(C)**.

**Figure 5:**
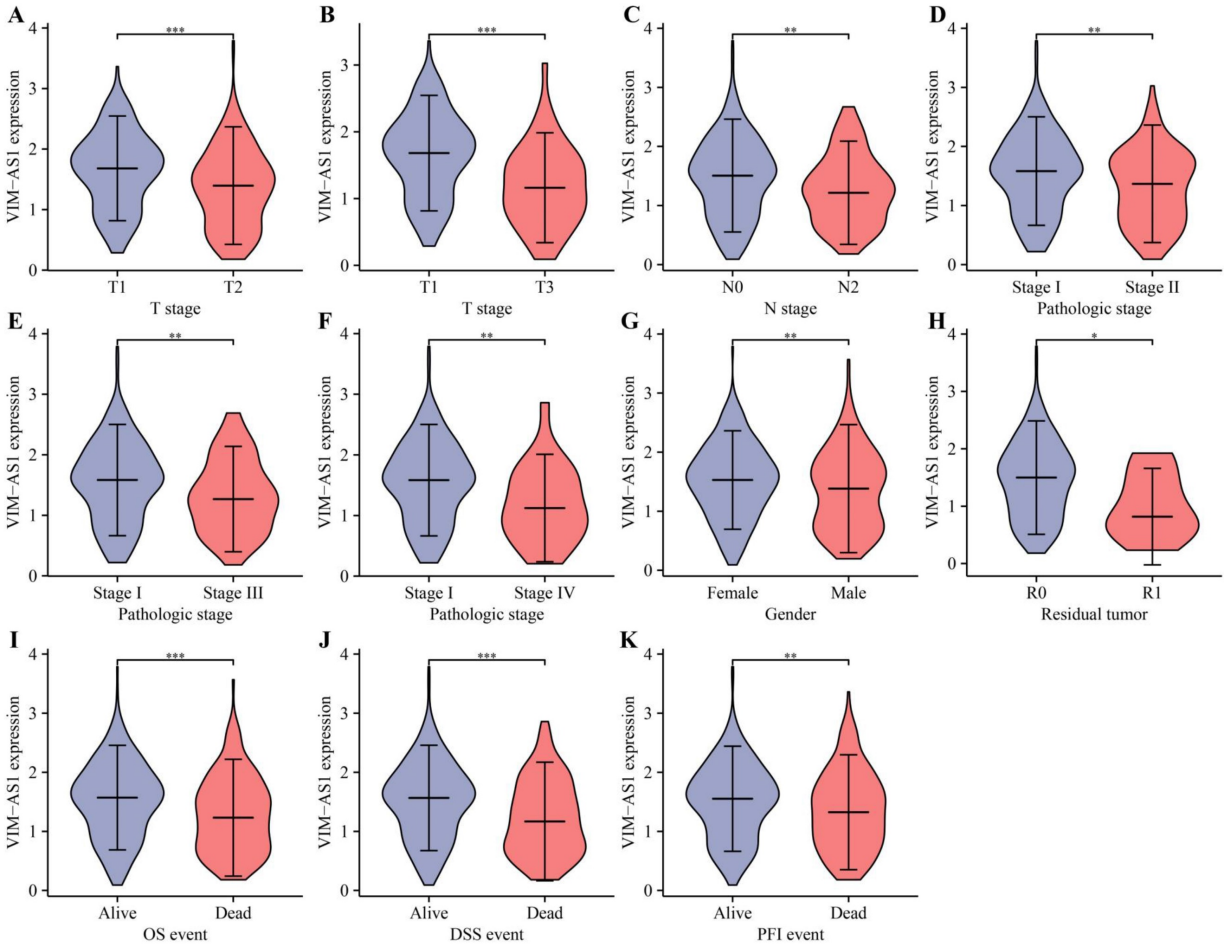
Correlation between VIM-AS1 expression and clinical parameters includes pathological and TNM stages. **(A-B)** T-stage; **(C)** lymph node metastasis; **(D-F)** pathological stage; **(G)** gender; **(H)** residual tumor;** (I)** OS; **(J)** DSS; **(K)** PFI. *P<0.05, **P<0.01, ***P<0.001, ****P<0.0001.

Kaplan-Meier analysis revealed that low levels of VIM-AS1 expression were significantly associated with adverse overall survival (OS), disease-free survival (DFS), and progression-free survival (PFS) (**Figure 4**). We then explored the correlations between VIM-AS1 expression and clinical parameters, including TNM stage, pathologic stage, and residual tumor. We found that VIM-AS1 expression was significantly correlated with pathological and TNM stages (**Figure 5**).

We furtherly tested the prognostic performance of VIM-AS1 in subgroups of LUAD patients. All patients were divided into various subgroups based on their clinical features. Survival analyses performed in the subgroups indicated that VIM-AS1 performed well in subgroups (**Figures S1-3**). From above results, VIM-AS1 has promising prognostic values for LUAD patients.

### The prognostic significance of VIM-AS1 in lung adenocarcinoma

**Figure 6:**
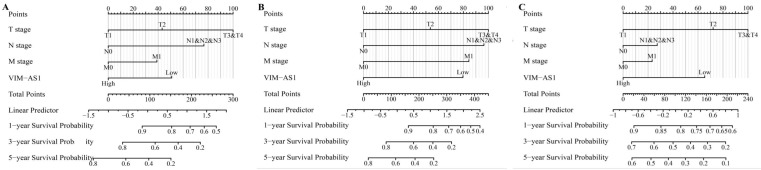
Construction and performance validation of the VIM-AS1-based nomogram for **(A)** OS; **(B)** DSS; **(C)** PFI in LUAD patients.

The multivariate COX regression analysis was conducted to determine whether the VIM-AS1 expression level and pathologic stage might be valuable prognostic biomarkers in LUAD. Our results showed that T stage, N stage, distant metastasis, pathological stage were adverse factors affecting OS and DSS in LUAD patients. Low VIM-AS1 expression level was an independent prognostic factor for LUAD patients (**Tables 1-3**). We established a nomogram to integrate VIM-AS1 as a LUAD biomarker. The prediction models were tested for overall survival, disease-free survival, and progression-free survival. These results showed that lower total points on the nomogram for OS, PFS, and DFS indicated a worse prognosis (**Figure 6**). Taken together, our findings indicated that VIM-AS1 could well predict clinical outcomes of lung adenocarcinoma patients.

### Biological functions of VIM-AS1 co-expressed genes

**Figure 7:**
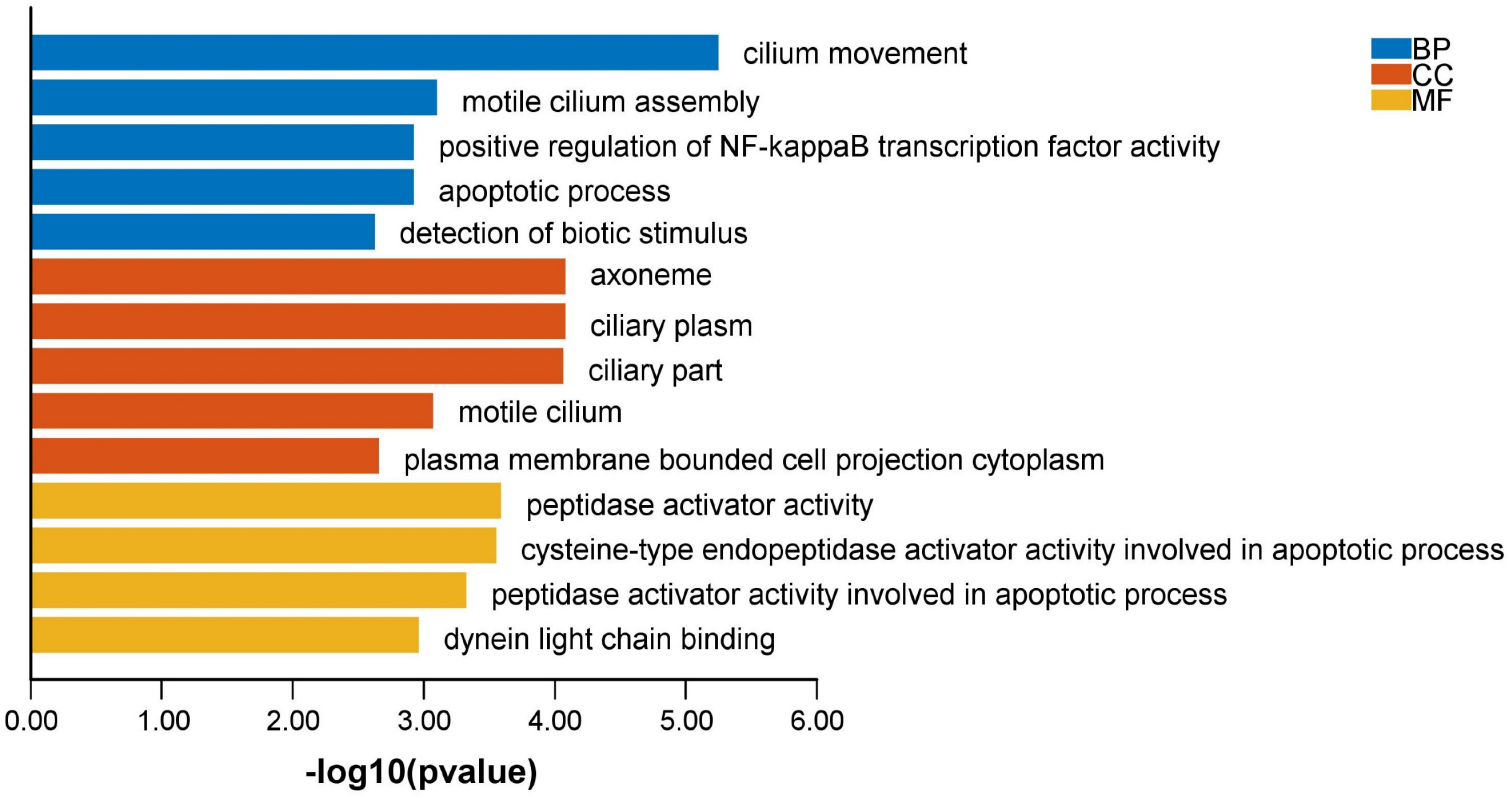
Biological functions of VIM-AS1 co-expressed genes.

To explore the influence of potential signaling pathways of LncVIM-AS1. We filtered 214 VIM-AS1 co-expressed genes by correlation analysis (**Table S2**). GO annotation showed that VIM-AS1 co-expressed genes were involved in cilium movement, positive regulation of NF-kappaB transcription factor activity, apoptotic process. Moreover, the cysteine-type endopeptidase activator activity involved in apoptotic process is also enriched in molecular function analysis (**Figure 7**).

### VIM-AS1 regulates apoptosis process in A549 cell

**Figure 8:**
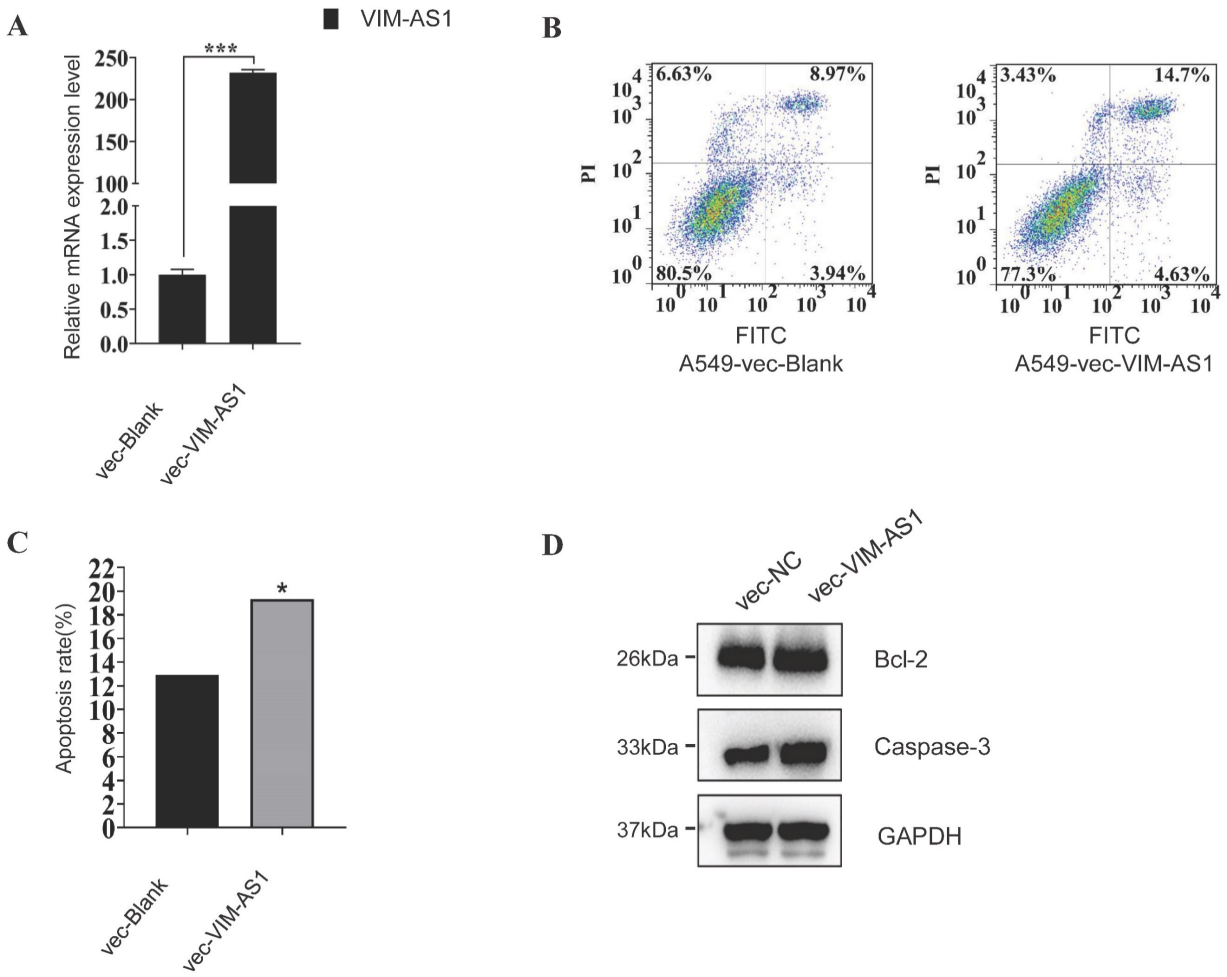
VIM-AS1 overexpression influences A549 cell apoptosis. **(A-C)** Up-regulating lncVIM-AS1 increases the apoptosis rate in A549 detected by FCM. **(D)** Apoptosis-related proteins are tested by Western blot. * *P*<0.05, ***P<0.001.

These enrichment results suggested that lncRNA might regulate cell apoptosis in LUAD, so we further testified whether VIM-AS1 impacts apoptosis in lung cancer. We overexpressed the VIM-AS1 mRNA in A549, the classical lung adenocarcinoma cell line. We found the apoptosis rate significantly increase when the VIM-AS1 was up-regulated (blank is 12.91%, vecVIM-AS1 is 19.33%) (**Figure 8**). Also, we found the apoptosis-related protein BCl_2_ and Caspase-3 when the lncVIM-AS1 was overexpression in A549.

## Discussion

Long non-coding RNAs (lncRNAs), which are defined as RNAs longer than 200 nucleotides in length. Some of these play crucial roles in extensive biological processes such as epigenetic regulation, transcriptional regulation, cell growth, and tumorigenesis 11, 12. In the decades, studies reveal the importance of lncRNAs for variable and complex development and progression in cancer 13. Moreover, lncRNAs act as key regulators of gene expression in many different cellular pathways and systems. Understanding the roles that lncRNA is involved in can not only help us find new therapeutic targets but also provide early clues about tumor development.

Since lncRNAs take crucial effects on tumor development, they are used as biomarkers for predicting and treating lung adenocarcinoma. For example, metastasis-associated lung adenocarcinoma transcript 1 (MALAT1) has been widely studied in cancer lung adenocarcinoma. MALAT1 associated cancer signaling pathways include MAPK/ERK, β-catenin/Wnt, PI3K/AKT. These pathways are involved in multiple steps in the development of tumors. Additionally, the diagnostic and prognostic significance of MALAT1 has been demonstrated in lung adenocarcinoma 14. Although a variety of lncRNAs have been found significant roles in the mechanisms of lung cancer, considering that lncRNAs are variable and complex, thus it is necessary to explore more diverse lncRNA mechanisms in lung cancer.

To collect lncRNA expression data from TCGA and GTEx, we found that VIM-AS1 showed the differences between lung tumor and normal tissues, with significantly lower expression in lung tumor tissues. In other cancers, VIM-AS1 has been reported different expression features. For example, VIM-AS1 expression levels are significantly downregulated in breast cancer tissues 15, while elevated in oral carcinoma 16. In these studies, the expression of VIM-AS1 also showed promising diagnostic power for discrimination of malignancy tissues from normal tissues. Moreover, VIM-AS1 was regarded as an independent diagnostic biomarker in tumors and its expression levels were associated with cancer metastasis and poor patient prognosis, including bladder cancer, colorectal cancer, and glioma 16, 17. In early studies, VIM-AS1 has been reported to be differentially expressed between lung cancer tissues and normal tissues 18. However, because the sample size of the study was limited and the analysis method was not systematic enough, we conducted a more in-depth analysis for the clinical diagnostic value of VIM-AS1. We found patients with tumors characterized by low VIM-AS1 expression had markedly worse OS, DSS, and PFI by Kaplan-Meier analysis. And we also determined that more advanced tumor grade and stage was associated with lower VIM-AS1 expression level. In addition, VIM-AS1 showed significant prognosis value in subtypes lung cancer, which has not been reported yet. The COX regression analysis showed that VIM-AS1 expression level decreased as an independent influencing factor of poor prognosis in LUAD patients. VIM-AS1 nomogram and risk models are expected to assess the prognosis of LUAD patients. In summary, we believe VIM-AS1 can be a potential biomarker to predict the prognosis of lung adenocarcinoma.

Many studies have reported VIM-AS1 is involved in the mechanisms of malignant tumor progression. In colorectal cancer, VIM-AS1 upregulates in high-grade, lymph node metastatic, and vascular invasive tumors 8. Downregulating VIM-AS1 expression levels can inhibit cell cycle and proliferation by promoting apoptosis and cellular senescence. VIM-AS1 inducing the epithelial-mesenchymal transition process is the crucial step to promote tumor growth and metastasis 17. In prostate cancer, VIM-AS1 was significantly overexpressed in prostate cancer tissues. High VIM-AS1 expression levels are correlated with tumor size, metastasis, and TNM stages. When VIM-AS1 expression was inhibited, the abilities of PC3 cell proliferation, migration, and invasion were weakened 19. At present, there is still a lack of studies to prove the value of VIM-AS1 in lung cancer.

As for lung adenocarcinoma, VIM-AS1 was regarded as an immune-related lncRNA, which indicated regulating immune response might be a potential mechanism of tumor progression 20. But this hypothesis remains lacks strong experimental evidence. Therefore, we tend to explore the mechanisms of VIM-AS1 in lung adenocarcinoma. Firstly, we filter the co-expression genes based on VIM-AS1, then we conducted the enrichment analyses to explain those genes' molecular functions. We found VIM-AS1 was enriched for some tumor progression-related biofunctions, such as NF-kappaB and apoptosis process. A wide variety of malignancies commonly exhibit aberrant NF-kappaB constitutive expression in human which results in tumorigenic processes and cancer survival in a variety of lung tumors 21, 22. NF-kappaB signaling mechanism is mainly involved in the progression of lung tumorigenesis. Thus, tremendous research in designing a variety of NF-kappaB antagonists is undergoing 23. The “apoptosis process” is also the top enrichment term in our results. Apoptosis has been investigated in the past decades and has been regarded as the major mechanism of development and programmed cell death 24. The processes of apoptosis induce cell death upon cellular stress, which is crucial in the pathophysiology of lung cancer 25. To prove the importance of VIM-AS1, we constructed the A549 with VIM-AS1 overexpression. The results indicated that VIM-AS1 expression can promote cell apoptosis. In previous study, VIM-AS1 binding miR-105-5p is a potential mechanism for enhancing glioma cell apoptosis 9. However, how VIM-AS1 regulates tumor cell apoptosis is barely known. In the study of gastric cancer, VIM‑AS1 activated the Wnt/β‑catenin pathway might be associated with apoptosis of AGS and HGC‑27 cells, but it also lacks direct evidence 26. In recent studies, VIM-AS1 has been found to regulate EMT (epithelial-mesenchymal transition) 8, 27. As we know, EMT is a key process in cancer cell metastasis, during which epithelial cell acquire mesenchymal characteristics, including enhanced cell motility and migration. Besides, malignant tumor cells must overcome these various forms of cell death (including apoptosis) to metastasize 28. Therefore, we speculate that V may affect apoptotic signaling by regulating the underlying EMT mechanism. However, our results provide significant evidence to indicate the relationship between VIM-AS1 and apoptosis, the more advanced researches also need to conduct.

A large sample of TCGA database was utilized to find that VIM-AS1 has an important role in LUAD progression. This requires more investigators to verify the expression of VIM-AS1 and the effect on cell growth and migration by collecting LUAD tissues and cellular experiments. Combined with the reports in the literature, VIM-AS1 deserves to be investigated in depth regarding the mechanism of LUAD cell metastasis. Overall, VIM-AS1 expression levels were significantly downregulated in LUAD tissues and significantly associated with short OS, DSS, significant PFI, and pathological staging, lymph node metastasis, Male and complete resection in LUAD patients. Decreased VIM-AS1 expression levels were an independent risk factor for poor prognosis in LUAD patients.VIM-AS1 co-expressed genes constructed by VIM-AS1 expression were significantly associated with the immune microenvironment in LUAD. The VIM-AS1 nomogram and risk models are expected to be a tool for assessing the prognosis of LUAD patients.

This study offers more understanding of the correlation between VIM-AS1 and LUAD, but some limitations still exist. First, although we validated the expression level of VIM-AS1 in patients, there is a lack of enough samples to make results more solid. Secondly, we proved VIM-AS1 had significant roles in A549, but more direct shreds of evidence need to be explored in further studies to explain the mechanism of apoptosis which is involved in VIM-AS1. Furthermore, we will perform more *in vivo* and *in vitro* experiments to explore the potential molecular mechanisms of VIM-AS1 in tumor metastasis and tumor microenvironment regulation of LUAD.

## Supplementary Material

Supplementary figures and tables.

## Figures and Tables

**Table 1:** Prognosis analysis of overall survival in clinical LUAD cohort

Characteristics	N	HR (95% CI)	P	HR (95% CI)	P
T stage	523				
T1	175	Reference			
T2	282	1.521 (1.068-2.166)	0.02	1.580 (1.003-2.489)	0.049
T3	47	2.937 (1.746-4.941)	<0.001	3.236 (1.591-6.585)	0.001
T4	19	3.326 (1.751-6.316)	<0.001	2.045 (0.931-4.490)	0.075
N stage	510				
N0	343	Reference			
N1	94	2.381 (1.695-3.346)	<0.001	2.227 (1.167-4.249)	0.015
N2	71	3.108 (2.136-4.521)	<0.001	1.980 (0.826-4.746)	0.126
N3	2	0.000 (0.000-Inf)	0.994	0.000 (0.000-Inf)	0.993
M stage	377				
M0	352	Reference			
M1	25	2.136 (1.248-3.653)	0.006	1.773 (0.808-3.891)	0.153
Pathologic stage	518				
Stage I	290	Reference			
Stage II	121	2.418 (1.691-3.457)	<0.001	0.896 (0.449-1.791)	0.757
Stage III	81	3.544 (2.437-5.154)	<0.001	1.420 (0.537-3.759)	0.48
Stage IV	26	3.790 (2.193-6.548)	<0.001		
Gender	526				
Female	280	Reference			
Male	246	1.070 (0.803-1.426)	0.642		
Age	516				
<=65	255	Reference			
>65	261	1.223 (0.916-1.635)	0.172		
Smoker	512				
No	72	Reference			
Yes	440	0.894 (0.592-1.348)	0.591		
VIM-AS1	526				
Low	260	Reference			
High	266	0.577 (0.431-0.773)	<0.001	0.613 (0.432-0.868)	0.006

**Table 2:** Prognosis analysis of DSS in clinical LUAD cohort

Characteristics	N	HR (95% CI)	P	HR (95% CI)	P
T stage	488				
T1	168	Reference			
T2	262	1.701 (1.085-2.668)	0.021	1.519 (0.853-2.705)	0.156
T3	43	2.846 (1.453-5.572)	0.002	2.563 (0.977-6.726)	0.056
T4	15	2.770 (1.061-7.230)	0.037	1.805 (0.582-5.597)	0.306
N stage	475				
N0	327	Reference			
N1	83	2.751 (1.808-4.185)	<0.001	2.110 (0.925-4.809)	0.076
N2	63	2.762 (1.698-4.493)	<0.001	1.916 (0.551-6.666)	0.307
N3	2	0.000 (0.000-Inf)	0.995	0.000 (0.000-Inf)	0.996
M stage	344				
M0	323	Reference			
M1	21	2.455 (1.269-4.749)	0.008	2.279 (0.922-5.633)	0.074
Pathologic stage	483				
Stage I	277	Reference			
Stage II	112	3.017 (1.931-4.715)	<0.001	1.090 (0.447-2.658)	0.849
Stage III	72	3.326 (2.028-5.457)	<0.001	1.292 (0.325-5.143)	0.716
Stage IV	22	4.632 (2.371-9.050)	<0.001		
Gender	491				
Female	262	Reference			
Male	229	0.989 (0.687-1.424)	0.954		
Age	481				
<=65	243	Reference			
>65	238	1.013 (0.701-1.464)	0.944		
Smoker	477				
No	69	Reference			
Yes	408	1.040 (0.602-1.796)	0.889		
VIM-AS1	491				
Low	240	Reference			
High	251	0.495 (0.340-0.721)	<0.001	0.523 (0.329-0.832)	0.006

**Table 3:** Prognosis analysis of PFI in clinical LUAD cohort

Characteristics	N	HR (95% CI)	P	HR (95% CI)	P
T stage	523				
T1	175	Reference			
T2	282	1.758 (1.276-2.422)	<0.001	1.515 (1.090-2.105)	0.013
T3	47	3.495 (2.199-5.556)	<0.001	2.450 (1.332-4.508)	0.004
T4	19	1.113 (0.444-2.791)	0.819	0.505 (0.166-1.534)	0.228
N stage	510				
N0	343	Reference			
N1	94	1.540 (1.118-2.122)	0.008	1.215 (0.691-2.136)	0.499
N2	71	1.498 (1.018-2.205)	0.04	0.510 (0.178-1.465)	0.211
N3	2	0.906 (0.127-6.485)	0.922	0.781 (0.088-6.921)	0.825
M stage	377				
M0	352	Reference			
M1	25	1.513 (0.855-2.676)	0.155		
Pathologic stage	518				
Stage I	290	Reference			
Stage II	121	2.013 (1.478-2.742)	<0.001	1.298 (0.734-2.297)	0.37
Stage III	81	1.831 (1.257-2.669)	0.002	2.872 (0.940-8.769)	0.064
Stage IV	26	2.086 (1.189-3.657)	0.01	2.006 (1.010-3.985)	0.047
Gender	526				
Female	280	Reference			
Male	246	1.172 (0.901-1.526)	0.236		
Age	516				
<=65	255	Reference			
>65	261	1.023 (0.784-1.335)	0.867		
Smoker	512				
No	72	Reference			
Yes	440	0.968 (0.658-1.426)	0.87		
VIM-AS1	526				
Low	260	Reference			
High	266	0.646 (0.496-0.841)	0.001	0.761 (0.577-1.004)	0.053

## Abbreviations

LUAD: lung adenocarcinoma
EMT: epithelial mesenchymal transition
OS: overall survival
DSS: disease-specific survival
PFI: progress free interval
TCGA: Cancer Genome Atlas
GTEx: genotypic tissue expression
GO: Gene Ontology
BP: biological processes
CC: cellular components
MF: molecular functions
