# Supplementary material for: lncRNA VIM-AS1 acts as a prognostic biomarker and promotes apoptosis in lung adenocarcinoma
Source: J Cancer. 2023 May 15;14(8):1417–26. doi: 10.7150/jca.83639 (PMC10240666; doi:10.7150/jca.83639)
Supplement: Supplementary file 1 — Supplementary figures and tables. [file jcav14p1417s1.pdf]

Figure S1. Kaplan–Meier survival analyses for overall survival of VIM-AS1 in different subgroups stratified by clinical features.

Figure S2. Kaplan–Meier survival analyses for disease-specific survival of VIM-AS1 in different subgroups stratified by clinical features.

Figure S3. Kaplan–Meier survival analyses for progression-free survival of VIM-AS1 in different subgroups stratified by clinical features.

Figure S1

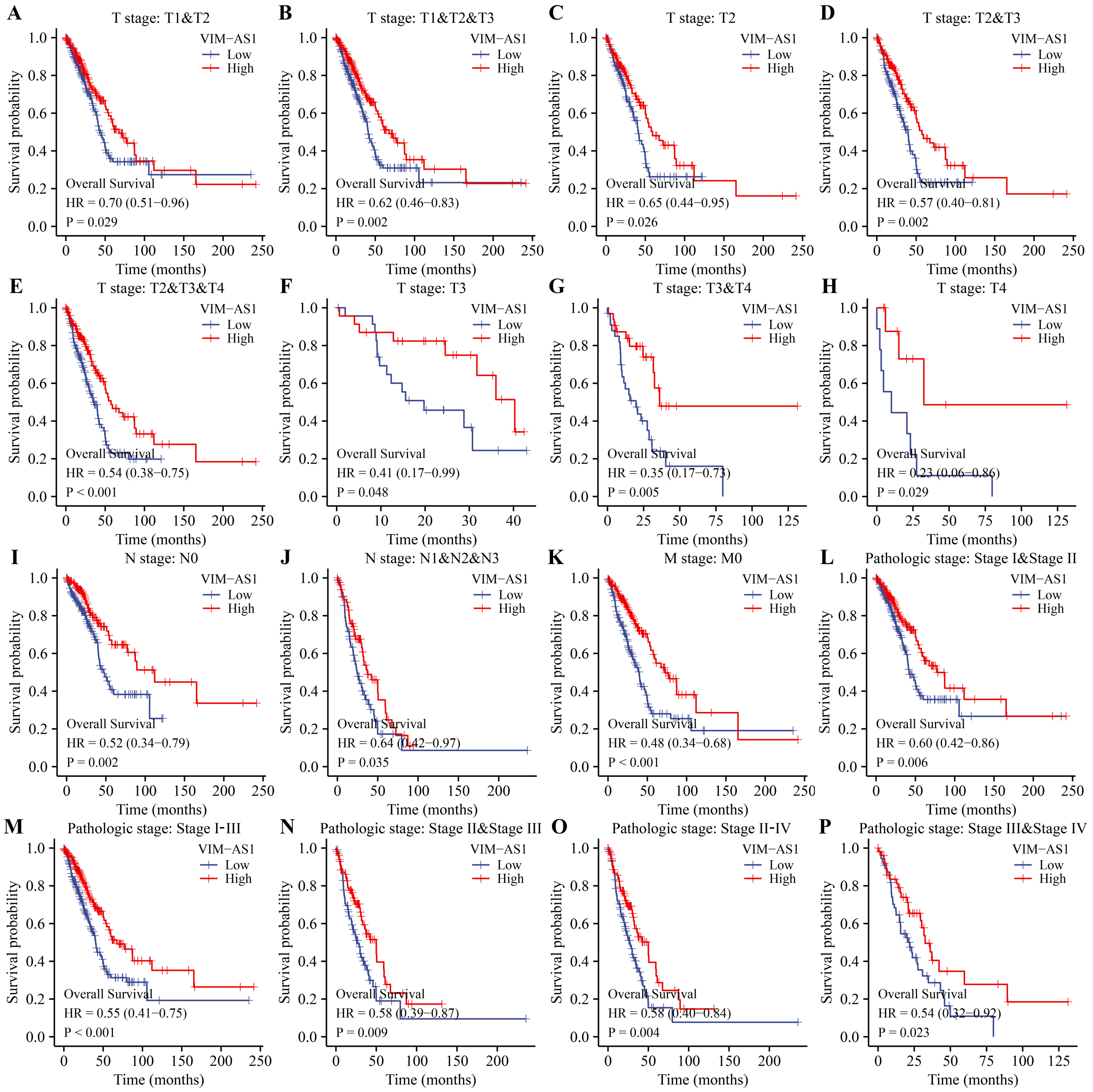
A
T stage: T1&T2
VIM−AS1
Low
High
Overall Survival
HR = 0.70 (0.51−0.96)
P = 0.029
B
T stage: T1&T2&T3
Overall Survival
HR = 0.62 (0.46−0.83)
P = 0.002
C
T stage: T2
Overall Survival
HR = 0.65 (0.44−0.95)
P = 0.026
D
T stage: T2&T3
Overall Survival
HR = 0.57 (0.40−0.81)
P = 0.002
E
T stage: T2&T3&T4
Overall Survival
HR = 0.54 (0.38−0.75)
P < 0.001
F
T stage: T3
Overall Survival
HR = 0.41 (0.17−0.99)
P = 0.048
G
T stage: T3&T4
Overall Survival
HR = 0.35 (0.17−0.73)
P = 0.005
H
T stage: T4
Overall Survival
HR = 0.23 (0.06−0.86)
P = 0.029
I
N stage: N0
Overall Survival
HR = 0.52 (0.34−0.79)
P = 0.002
J
N stage: N1&N2&N3
Overall Survival
HR = 0.64 (0.42−0.97)
P = 0.035
K
M stage: M0
Overall Survival
HR = 0.48 (0.34−0.68)
P < 0.001
L
Pathologic stage: Stage I&Stage II
Overall Survival
HR = 0.60 (0.42−0.86)
P = 0.006
M
Pathologic stage: Stage I-III
Overall Survival
HR = 0.55 (0.41−0.75)
P < 0.001
N
Pathologic stage: Stage II&Stage III
Overall Survival
HR = 0.58 (0.39−0.87)
P = 0.009
O
Pathologic stage: Stage II-IV
Overall Survival
HR = 0.58 (0.40−0.84)
P = 0.004
P
Pathologic stage: Stage III&Stage IV
Overall Survival
HR = 0.54 (0.32−0.92)
P = 0.023
Survival probability
Time (months)

Figure S2

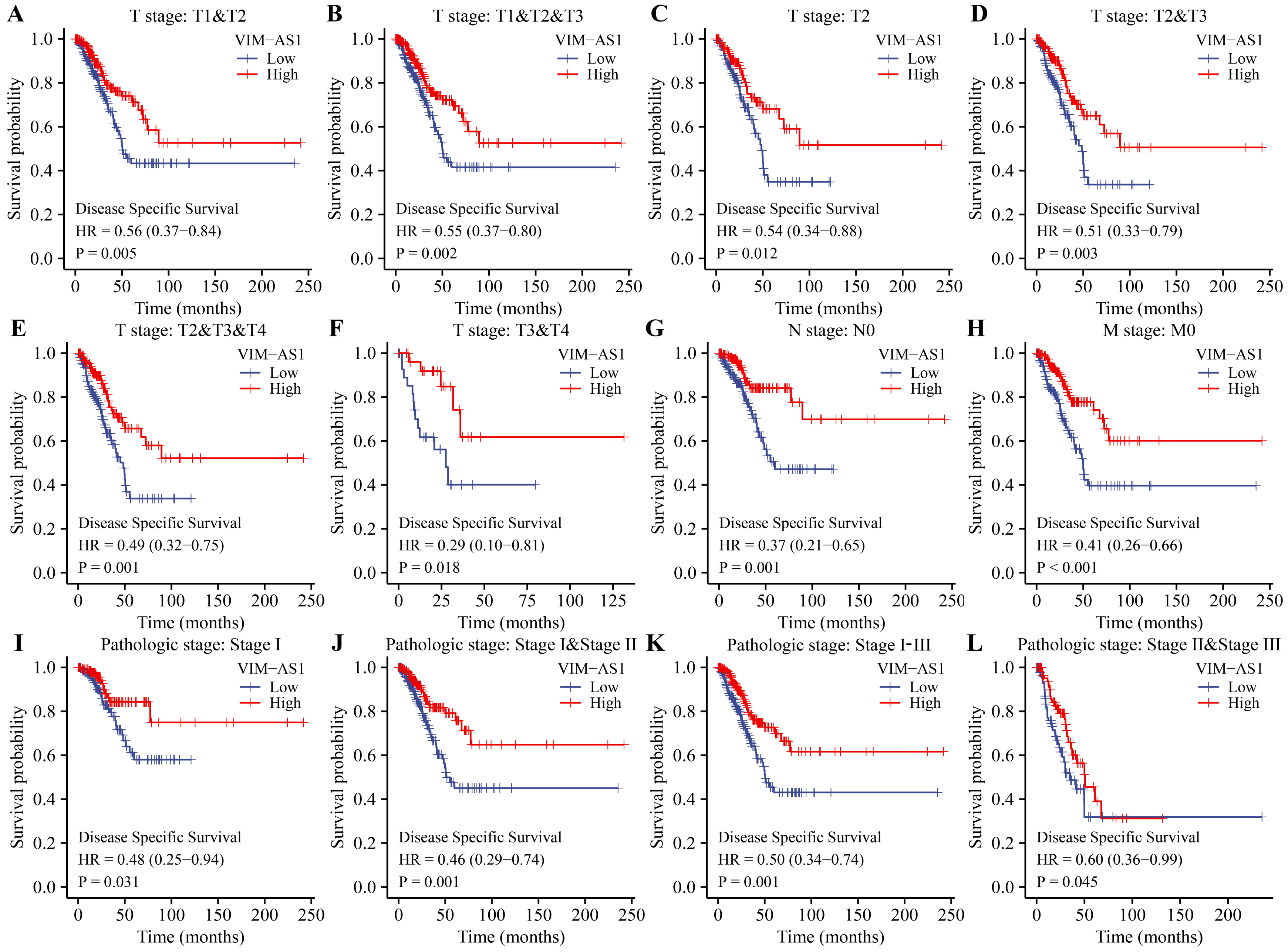
A
T stage: T1&T2
VIM−AS1
Low
High
Survival probability
Disease Specific Survival
HR = 0.56 (0.37−0.84)
P = 0.005
Time (months)
B
T stage: T1&T2&T3
Disease Specific Survival
HR = 0.55 (0.37−0.80)
P = 0.002
C
T stage: T2
Disease Specific Survival
HR = 0.54 (0.34−0.88)
P = 0.012
D
T stage: T2&T3
Disease Specific Survival
HR = 0.51 (0.33−0.79)
P = 0.003
E
T stage: T2&T3&T4
Disease Specific Survival
HR = 0.49 (0.32−0.75)
P = 0.001
F
T stage: T3&T4
Disease Specific Survival
HR = 0.29 (0.10−0.81)
P = 0.018
G
N stage: N0
Disease Specific Survival
HR = 0.37 (0.21−0.65)
P = 0.001
H
M stage: M0
Disease Specific Survival
HR = 0.41 (0.26−0.66)
P < 0.001
I
Pathologic stage: Stage I
Disease Specific Survival
HR = 0.48 (0.25−0.94)
P = 0.031
J
Pathologic stage: Stage I&Stage II
Disease Specific Survival
HR = 0.46 (0.29−0.74)
P = 0.001
K
Pathologic stage: Stage I-III
Disease Specific Survival
HR = 0.50 (0.34−0.74)
P = 0.001
L
Pathologic stage: Stage II&Stage III
Disease Specific Survival
HR = 0.60 (0.36−0.99)
P = 0.045

Figure S3

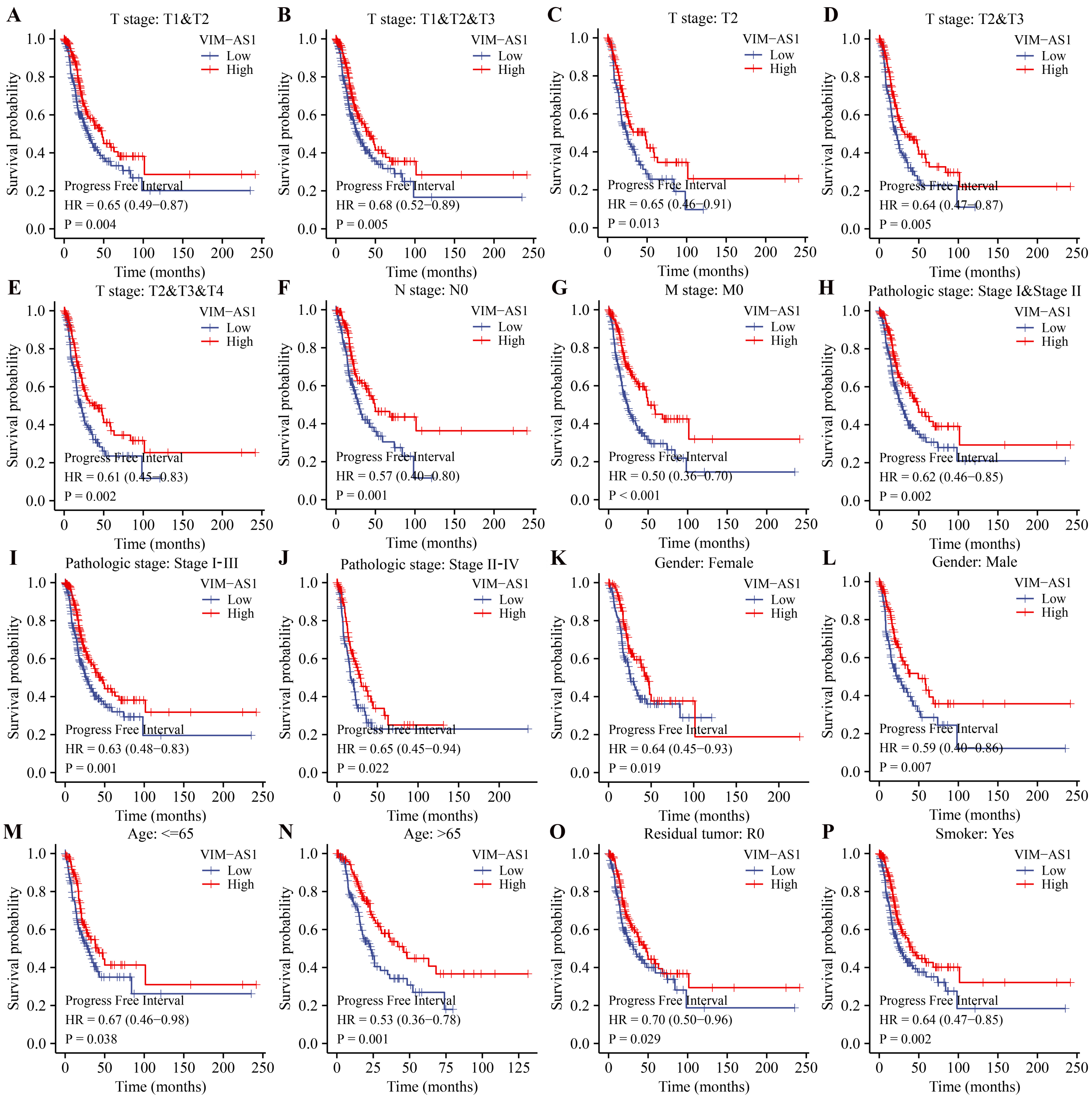

A
T stage: T1&T2
HR = 0.65 (0.49−0.87)
P = 0.004
B
T stage: T1&T2&T3
HR = 0.68 (0.52−0.89)
P = 0.005
C
T stage: T2
HR = 0.65 (0.46−0.91)
P = 0.013
D
T stage: T2&T3
HR = 0.64 (0.47−0.87)
P = 0.005
E
T stage: T2&T3&T4
HR = 0.61 (0.45−0.83)
P = 0.002
F
N stage: N0
HR = 0.57 (0.40−0.80)
P = 0.001
G
M stage: M0
HR = 0.50 (0.36−0.70)
P < 0.001
H
Pathologic stage: Stage I&Stage II
HR = 0.62 (0.46−0.85)
P = 0.002
I
Pathologic stage: Stage I-III
HR = 0.63 (0.48−0.83)
P = 0.001
J
Pathologic stage: Stage II-IV
HR = 0.65 (0.45−0.94)
P = 0.022
K
Gender: Female
HR = 0.64 (0.45−0.93)
P = 0.019
L
Gender: Male
HR = 0.59 (0.40−0.86)
P = 0.007
M
Age: <=65
HR = 0.67 (0.46−0.98)
P = 0.038
N
Age: >65
HR = 0.53 (0.36−0.78)
P = 0.001
O
Residual tumor: R0
HR = 0.70 (0.50−0.96)
P = 0.029
P
Smoker: Yes
HR = 0.64 (0.47−0.85)
P = 0.002
VIM−AS1
Low
High
Progress Free Interval
Survival probability
Time (months)

Table S1 Basic clinical information of the patients

Number	Age	sex(M/F)	Tumor location	Tumor type	Tumor size(cm)	Lymphatic metastasis(Y/N)	Stage	Grade
1#	41	F	right-up	Minimally invasive adenocarcinoma	1.4	N	T1a	Ia
2#	67	F	right-up	Lung Adenocarcinoma， acinar	1.7	N	T1a	Ia
3#	56	M	left-up	Lung Adenocarcinoma， acinar	3	N	T1b	Ia
4#	76	F	left-up	Lung Adenocarcinoma, papillary	1.9	N	T1a	Ia
5#	65	M	right-down	Lung Adenocarcinoma, papillary	4.6	Y	T2a	IIa
6#	68	M	right-up	Lung Adenocarcinoma, papillary	1.4	N	T1a	Ia
7#	59	M	right-up	Lung Adenocarcinoma, papillary	5.2	N	T2b	Ib
8#	72	M	left-up	Lung Adenocarcinoma, papillary	2.57	N	T1b	Ia
9#	69	F	right-down	Lung Adenocarcinoma， acinar	1.9	N	T1a	Ia
10#	50	F	right-medium	Lung Adenocarcinoma, papillary	2.5	N	T1b	Ia
11#	76	F	right-up	Lung Adenocarcinoma, papillary	2.6	N	T1b	Ia
12#	52	F	right-up	Lung Adenocarcinoma， acinar	1.6	N	T1a	Ia

TableS2 Table S2 VIM-AS1 co-expressed genes

Gene markers	*Cor*	*P*
HLA-DPB1	0.348	<0.001
HLA-DRA	0.347	<0.001
CCR7	0.337	<0.001
CD1C	0.337	<0.001
HLA-DPA1	0.335	<0.001
HLA-DQB2	0.319	<0.001
STAT4	0.318	<0.001
HLA-DQB1	0.309	<0.001
CD2	0.302	<0.001
CTLA4	0.302	<0.001
ITGAX	0.282	<0.001
CD19	0.273	<0.001
CD3E	0.261	<0.001
NRP1	0.255	<0.001
CD3D	0.243	<0.001
STAT5A	0.242	<0.001
IRF5	0.236	<0.001
TBX21	0.225	<0.001
CD8A	0.206	<0.001
BCL6	0.206	<0.001
CD86	0.205	<0.001
ITGAM	0.201	<0.001
HAVCR2	0.197	<0.001
TNF	0.196	<0.001
CSF1R	0.19	<0.001
IL13	0.184	<0.001
IFNG	0.174	<0.001
CCL2	0.173	<0.001
PDCD1 (PDCD1)	0.172	<0.001
IL10	0.171	<0.001
CD79A	0.17	<0.001
CCR8	0.17	<0.001
FOXP3	0.153	<0.001
IL21	0.145	<0.001
MS4A4A	0.131	0.002
CEACAM8	0.127	0.003
CD8B	0.121	0.005
LAG3	0.107	0.013
CD163	0.106	0.015
GATA3	0.092	0.034
HLA-DQB3	0.091	0.035

IL17A	0.088	0.043
VSIG4	0.086	0.046
STAT5B	0.085	0.05
STAT1	0.075	0.084
STAT6	0.071	0.102
GZMB	0.033	0.441
CD68	0.024	0.582
PTGS2	0.021	0.622
KIR2DS4	-0.027	0.526
KIR3DL1	-0.031	0.467
NOS2	-0.041	0.346
KIR3DL3	-0.05	0.252
KIR2DL4	-0.053	0.224
KIR2DL3	-0.056	0.199
KIR3DL2	-0.09	0.037
KIR2DL1	-0.119	0.006
STAT3	-0.13	0.003
